# The Macroevolutionary Consequences of Niche Construction in Microbial Metabolism

**DOI:** 10.3389/fmicb.2021.718082

**Published:** 2021-10-04

**Authors:** Djordje Bajić, María Rebolleda-Gómez, Martha M. Muñoz, Álvaro Sánchez

**Affiliations:** ^1^ Department of Ecology and Evolutionary Biology, Yale University, New Haven, CT, United States; ^2^ Microbial Sciences Institute, Yale University, West Haven, CT, United States; ^3^ Department of Ecology and Evolutionary Biology, University of California Irvine, Irvine, CA, United States

**Keywords:** macroevolution, metabolism, innovation, diversification, niche construction, genotype-phenotype (G-P) map, genotype-by-environment (G×E) interaction, non-commutative epistasis

## Abstract

Microorganisms display a stunning metabolic diversity. Understanding the origin of this diversity requires understanding how macroevolutionary processes such as innovation and diversification play out in the microbial world. Metabolic networks, which govern microbial resource use, can evolve through different mechanisms, e.g., horizontal gene transfer or *de novo* evolution of enzymes and pathways. This process is governed by a combination of environmental factors, selective pressures, and the constraints imposed by the genetic architecture of metabolic networks. In addition, many independent results hint that the process of niche construction, by which organisms actively modify their own and each other’s niches and selective pressures, could play a major role in microbial innovation and diversification. Yet, the general principles by which niche construction shapes microbial macroevolutionary patterns remain largely unexplored. Here, we discuss several new hypotheses and directions, and suggest metabolic modeling methods that could allow us to explore large-scale empirical genotype-phenotype-(G-P)-environment spaces in order to study the macroevolutionary effects of niche construction. We hope that this short piece will further stimulate a systematic and quantitative characterization of macroevolutionary patterns and processes in microbial metabolism.

## Introduction

Prokaryotes exhibit by far the most diverse collection of metabolisms on earth. Disentangling the mechanisms by which such diversity arises is paramount for understanding both the emergence of complex life and the structure and function of modern microbial ecosystems.

Our knowledge about the history of life on earth contains numerous examples suggesting that the process of niche construction might play a central role in diversification. An obvious one is the early appearance of autotrophic metabolism, which profoundly transformed the biosphere by generating complex, energy-rich carbon molecules and releasing oxygen to the atmosphere, creating new ecological opportunities (Schirrmeister et al., 2013; Chen et al., 2020). Many additional examples exist in nature, where niche construction has been observed to play important roles, from the early examples described by Darwin (1881) in his work on earthworms to diatoms or beavers (Odling-Smee et al., 2013). Although, theoretical work has anticipated numerous ways, in which niche construction might impact evolutionary outcomes (Laland et al., 1999; Silver and Di Paolo, 2006; Krakauer et al., 2009), most of these predictions remain empirically untested. From a general principles standpoint, many open questions remain: How does niche construction itself evolve, and what are the principles and mechanisms that govern it? How does it depend on the external environment, on the architecture of metabolic genotypes or on metabolic strategies? How does niche construction affect macroevolutionary processes such as innovation and diversification?

The short-term, microevolutionary consequences of niche construction have been extensively characterized, both theoretically and empirically (Laland et al., 1999; Odling-Smee et al., 2013). The most paradigmatic of such effects are eco-evolutionary dynamics, which arise because constructed environments (and their effects on selective pressures) depend, within certain limits, on the abundance of the organisms constructing them. This leads to a dynamic coupling between frequency and density-dependent selection, which occur in similar timescales. Because the nature of built environments in microbes is often determined by single genes or mutations, niche construction can link the fate of specific alleles to the current, instantaneous composition of a population (Sanchez and Gore, 2013; Chen et al., 2014). When several species are involved, niche construction can also combine with other ecological interactions to generate more complex phenomena, such as the coexistence of three or more species through intransitive interactions (e.g., rock-paper-scissors; Kerr et al., 2002).

In contrast to microevolution, the macroevolutionary consequences of niche construction, such as microbial innovation and diversification, have been less explored. A rare experimental example of a potentially macroevolutionary event is the appearance of aerobic citrate utilization in *Escherichia coli* in the Long Term Evolution Experiment (LTEE). Recent findings have shown that the two main mutations leading to this innovation, the aerobic expression of dctA and citT, were intimately linked to an eco-evolutionary interaction mediated by the release of metabolites to the environment (Bajić et al., 2018; de Visser et al., 2018). Furthermore, one of the main potentiating mutations that helped “prepare” the genetic background for the evolution of citrate use (*gltA*), likely achieved fixation because of its beneficial effect on acetate, a constructed niche (Quandt et al., 2015). These observations suggest that niche construction might play a key role in microbial metabolic diversification. More broadly, they showcase the potential of microbial experiments to illuminate the mechanisms and the genetic basis underlying macroevolutionary patterns.

At the same time, experiments also have important limitations. In the LTEE, only one out of 12 *E. coli* evolution lines gained the ability to use citrate, and did so only after ~30,000 generations (~20years of experiment). This illustrates that innovation and exploration of untapped ecological opportunities still depends on historically contingent, and thus rare, combinations of mutations (Blount et al., 2008). Correspondingly, “blind” evolutionary explorations of genotype space still require timescales approaching the limits of what is experimentally feasible, even for organisms with some of the shortest generation times on Earth.

A promising alternative is provided by genome-scale metabolic models, which offer us the possibility to rapidly explore large regions of metabolic genotype-environment space. Using genome-inferred metabolic networks, these models are able to quite accurately simulate the growth of real organisms *in silico*, providing us mechanistic insight into the function of biologically realistic genotype-phenotype-(G-P)-fitness maps. Genome-scale metabolic models have been already successfully applied, for instance, to gain insight into long-term phenotypic evolution in microbes (Plata et al., 2015), study the genomic basis of metabolic innovations (Barve and Wagner, 2013; Hosseini et al., 2015) and explore intriguing origin-of-life scenarios (Goldford et al., 2017). Beyond purely computational studies, genome-scale metabolic models have also proven a powerful tool for experiment design. An astonishing example was the recent obtention of an *E. coli* strain capable of autotrophic metabolism (Gleizer et al., 2019). This achievement used metabolic modeling to predict what new reactions might be needed by *E. coli* to acquire carbon fixation capabilities. Once these reactions were included, experimental evolution took care of integrating them in the regulatory network, allowing *E. coli* to start fixing CO_2_ and become autotrophic in a relatively short time. The potential niche construction consequences of such metabolic innovation are self-evident.

We thus believe that, in combination with experiments, genome-scale metabolic models can be an invaluable tool to explore macroevolutionary patterns in microbes. In this short piece, we lay out several future directions, focusing in particular on the effects of niche construction or, more broadly, the two-way interaction between genotype and environment.

## Niche Construction and the Parallel Exploration of Fitness Landscapes

In their landmark work almost 40years ago, Levins and Lewontin (1985) noted that evolution does not only proceed as a mere adaptation of organisms to the external environment. In addition, as they adapt, organisms also modify the environment, potentially affecting their own selective pressures (Levins and Lewontin, 1985). Evolution becomes then better described as a “dialectic” process, in which genotype and environment perpetually modify each other. This logic, however, challenges the predictions of many established theories that only consider the adaptation of organisms to the “external” environment. For instance, fitness landscapes have been widely used as both a conceptual device and as a tool for predicting evolution (de Visser et al., 2018; Gorter et al., 2018). But if fitness landscapes constantly “deform” during evolution, their utility would be severely compromised (Doebeli et al., 2017).

To what extent, then, is niche construction an ubiquitous process, and to what extent is it able to influence evolutionary patterns and outcomes? Answering these key questions inevitably requires turning our attention to empirical systems. In recent work, we used constraint-based metabolic modeling to systematically map the diversity of constructed niches on a metabolic genotype space, and their evolutionary consequences (Bajić et al., 2018; de Visser et al., 2018). We found that when a newly constructed niche becomes available, as a result of a mutation, multiple subsequent mutations (often epistatic to each other) are typically needed to take advantage of this new niche. This led to a surprising conclusion: while in the shorter term “static” fitness landscapes are typically predictive, the deformations gain importance as changes in both the environment and the population genotypes accumulate. In addition, recent studies have identified that large numbers of metabolites can be secreted by microbes, often at no cost (de Visser et al., 2018; Magdalena and Wagner, 2018; Pacheco et al., 2019). These results point to the possibility that niche construction might play a preeminent role in evolutionary processes that typically occur over longer timescales, possibly including macroevolutionary patterns such as phenotypic divergence and diversification.

**Figure 1:**
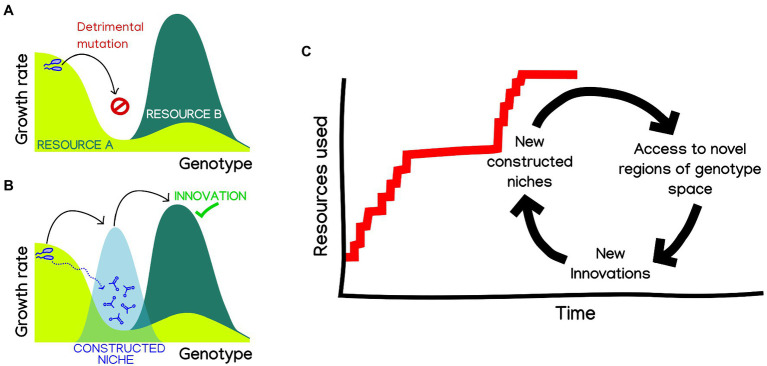
Niche construction could facilitate metabolic innovation and adaptive radiation in microbes. **(A)** Complex innovations, requiring two or more mutations, are hard to reach through adaptive evolution, needing the fixation of neutral or deleterious mutations. **(B)** Niche construction could provide “bridge” environments, which are known to facilitate the navigability of genotype space by adaptive evolution, making innovations readily accessible (Steinberg and Ostermeier, 2016; Szappanos et al., 2016; Pál and Papp, 2017). **(C)** By extension, we can imagine a hypothetical evolution of the number of resources that a microbial population can use, as a succession of innovation events. Changes in constructed niches could make new innovations accessible, which will spur adaptation and thus also building new niches. A hypothetical result of this cyclic process could be rapid bursts of innovation, a punctuated pattern characteristic of adaptive radiation.

A particularly interesting possibility is that, by enabling the parallel exploration of different fitness landscapes, niche construction could facilitate bridging fitness valleys (Steinberg and Ostermeier, 2016), including those leading to innovations. An illuminating hint of how this might happen comes from a recent work showing that the emergence of complex innovations can be facilitated by stepwise metabolic niche expansion (Szappanos et al., 2016). In order to reach complex innovations requiring two or more mutations, organisms capitalize on more accessible “stepping stone” innovations, allowing them to navigate genotype-space by switching between environments. It is easy to imagine how such “stepping-stones to innovation” could be provided through niche construction (Figure 1). In this way, niche construction could blur the lines between ecological and mutation-order speciation (Schluter, 2009), making them contingent on each other. Exploring to what extent can constructed niches open evolutionary paths toward otherwise inaccessible ecological opportunities could provide a mechanistic explanation to the hypothesis that “diversity beget diversity” (Whittaker, 1972), which has been recently shown to apply in microbiomes in some conditions (Madi et al., 2020).

## Linking Niche Construction to Adaptive Radiation

If niche construction facilitates innovation and diversification, this could have profound consequences for our understanding of adaptive radiations, a process considered integral to ecological and phenotypic diversity (Simpson, 1953; Schluter, 2000). Adaptive radiation is the process of phenotypic diversification of organisms into forms that fill different available ecological niches. Phenotypic novelties can facilitate adaptive radiation by allowing organisms to interact with their environments in new ways, in turn generating novel ecological opportunities upon which natural selection can act and prompting adaptive evolution (Simpson, 1953; Stroud and Losos, 2016; Erwin, 2017). The connection between innovation and adaptive radiation has been extensively documented both within the fossil record and using diverse empirical and experimental systems (Schluter, 2000; Losos, 2010; Yoder et al., 2010). Notable examples include the evolution of adhesive silk in spiders, which enhanced prey capture (Bond and Opell, 1998), alternate photosynthetic pathways in desert plants that reduce water loss (Silvestro et al., 2014), and adhesive toe pads that unlock access to arboreal niches in anole lizards (Burress and Muñoz, 2021). However, the study of ecological opportunity has been largely structured around the evolution of features that facilitate access to novel peaks in the adaptive landscape. Comparatively less focus has been given to the role that organisms serve as arbiters of available niches, for example, by constructing new ones or bridging access to new peaks (Emerson and Kolm, 2005; Erwin, 2005; Braakman et al., 2017). In the microbial realm, adaptive radiations of phenotypically diverse lineages can be obtained through experimental evolution, and often involve niche construction (Rainey and Travisano, 1998; Friesen et al., 2004; Le Gac et al., 2008; Kassen, 2009; Schick and Kassen, 2018). However, the scope of these studies in terms of environmental complexity, genetic diversity, and timescales is rather limited compared to plausible scenarios in nature (Consuegra et al., 2017).

A key question is to what extent are novelty, diversification and adaptive radiation in microbes constrained and directed by the spectrum of available genetic variation as opposed to just ecological opportunity (Schluter, 2009; Kassen, 2019). As shown by Schluter (1996), adaptive radiation often proceeds along “genetic lines of least resistance,” meaning that evolution occurs in the direction, where most genetic variation is available. If niche construction significantly alters microbial fitness landscapes, it could quantitatively bias the observed patterns of adaptive radiation by changing the distribution of fitness effects, and consequently altering the location of those lines of least resistance. Furthermore, complex genetic architecture (e.g., epistasis) also imposes strong constraints on adaptation (Weinreich et al., 2005, 2006), but this question has been scarcely explored in the context of adaptive radiation, and more generally, macroevolutionary patterns. In the case of bacterial metabolism, the availability of detailed empirical genotype-phenotype maps and the possibility of their prediction has already been very useful to show how the epistatic architecture of metabolic networks can fundamentally constrain innovation. For example, the partial overlapping between the pathways used for different nutrients results in many innovations being readily accessible as a byproduct to the adaptation to a given nutrient (Barve and Wagner, 2013). Exploring in detail how epistasis shapes the response of populations to constructed niches, and how epistatic interactions themselves change with the environment, represents an interesting opportunity for future research.

Furthermore, if niche construction provides “stepping stones” toward other innovations, it could also contribute qualitatively to some of the most iconic patterns of adaptive radiation such as rapid diversification. If we consider that each new adaptation bears the potential to transform the environment, we could imagine a “cycle” in which innovation results in new constructed environments, which in turn open up adaptive paths to further downstream innovations, potentially leading to rapid diversification patterns. Scenarios similar to the “stepping stones” model in adaptive radiation have been hinted at by simulations (Sneppen et al., 1995), but they have so far remained in the theoretical realm. Finally, an intriguing question is how niche construction plays out when considering more realistic genotype-phenotype spaces, where the genetic accessibility of phenotypes can be organized in asymmetric and nonrandom topologies (more precisely, “pre-topologies”; Fontana and Schuster, 1998; Stadler et al., 2001; Erwin, 2017). Together, exploring to what extent could niche construction facilitate innovation and release adaptive radiation from the yoke of genetic constraint is a fascinating future direction.

## Discussion

In this piece, we argued that the combination of genome-scale metabolic modeling with experiments presents a great opportunity to tackle the role of different evolutionary forces, and niche construction in particular, in microbial macroevolution. Recently, platforms such as “Computation of Microbial Ecosystems in Time and Space” (COMETS; Dukovski et al., 2020) are extending the range of possibilities of genome-scale metabolic models by enabling us to simulate evolution in the context of multispecies ecosystems. COMETS combines population dynamics with a realistic, empirically calibrated genotype-phenotype map that is also environment-sensitive, where mutations can randomly appear as either metabolic reaction deletions or additions (e.g., through horizontal gene transfer) or by random changes in the maximum fluxes through each reaction. Importantly, niche construction emerges naturally in COMETS, as it predicts phenotypes such as secretion of metabolites. This offers an unique opportunity to explore evolution (including macroevolution) with mechanistic insight, allowing us to understand biological processes at lower levels of organization without isolating them from the eco-evolutionary processes in which they are embedded (Bergelson et al., 2021; van Tatenhove-Pel et al., 2021). Furthermore, COMETS also offers sophisticated spatial capabilities. These could be key in understanding the effects of metabolic niche construction (Maynard et al., 2017; van Tatenhove-Pel et al., 2021) as well as help understand observations in natural environments, as well as in emerging experimental platforms such as ecoFABs (Sasse et al., 2019).

One of the hurdles in this path is our current lack of understanding of the relationship between the genotype and the organisms’ effects on the environment, particularly through secretion of metabolites. The release of some compounds, such as fermentation byproducts, is well understood (and predicted by metabolic models; Basan et al., 2015; Mori et al., 2016), suggesting that we could generalize this finding and link genotype to build niches in a systematic way. However, exometabolomic analyses typically identify complex metabolite mixtures (Paczia et al., 2012), whose origin is still poorly understood. Understanding the determinants of metabolic secretions represents one of the main current limitations for building a predictive theory of microbial ecology and evolution, including macroevolutionary processes of qualitative change.

## Data Availability Statement

The original contributions presented in the study are included in the article/supplementary material, further inquiries can be directed to the corresponding authors.

## Author Contributions

DB, MR-G, MM, and AS have contributed original ideas and perspectives presented in the manuscript. DB wrote the first draft. All authors contributed to the article and approved the submitted version.

## Funding

This work was supported by a young investigator award from the Human Frontier Science Program (RGY0077/2016), by a Packard Fellowship from the David and Lucile Packard foundation, and by the National Institutes of Health through grant 1R35 GM133467-01 to AS, and by a Templeton Foundation Grant (61866) to MM. MR-G acknowledges support from the Donnelly Fellowship from Yale University.

## Conflict of Interest

The authors declare that the research was conducted in the absence of any commercial or financial relationships that could be construed as a potential conflict of interest.

## Publisher’s Note

All claims expressed in this article are solely those of the authors and do not necessarily represent those of their affiliated organizations, or those of the publisher, the editors and the reviewers. Any product that may be evaluated in this article, or claim that may be made by its manufacturer, is not guaranteed or endorsed by the publisher.
